# The Cholinergic Anti-Inflammatory Pathway Delays TLR-Induced Skin Allograft Rejection in Mice: Cholinergic Pathway Modulates Alloreactivity

**DOI:** 10.1371/journal.pone.0079984

**Published:** 2013-11-21

**Authors:** Claude Sadis, Sophie Detienne, Benoît Vokaer, Louis-Marie Charbonnier, Philippe Lemaître, Chloé Spilleboudt, Sandrine Delbauve, Carole Kubjak, Véronique Flamand, Kenneth A. Field, Michel Goldman, Fleur S. Benghiat, Alain Le Moine

**Affiliations:** 1 Department of Anaesthesiology, Erasme Hospital, Brussels, Belgium; 2 Institute for Medical Immunology, Gosselies, Belgium; 3 Cell Biology/Biochemistry Program, Biology Department, Bucknell University, Lewisburg, Pennsylvania, United States of America; Centro Cardiologico Monzino IRCCS, Italy

## Abstract

Activation of innate immunity through Toll-like receptors (TLR) can abrogate transplantation tolerance by revealing hidden T cell alloreactivity. Separately, the cholinergic anti-inflammatory pathway has the capacity to dampen macrophage activation and cytokine release during endotoxemia and ischemia reperfusion injury. However, the relevance of the α7 nicotinic acetylcholine receptor (α7nAChR)-dependent anti-inflammatory pathway in the process of allograft rejection or maintenance of tolerance remains unknown. The aim of our study is to investigate whether the cholinergic pathway could impact T cell alloreactivity and transplant outcome in mice. For this purpose, we performed minor-mismatched skin allografts using donor/recipient combinations genetically deficient for the α7nAChR. Minor-mismatched skin grafts were not rejected unless the mice were housed in an environment with endogenous pathogen exposure or the graft was treated with direct application of imiquimod (a TLR7 ligand). The α7nAChR-deficient recipient mice showed accelerated rejection compared to wild type recipient mice under these conditions of TLR activation. The accelerated rejection was associated with enhanced IL-17 and IFN-γ production by alloreactive T cells. An α7nAChR-deficiency in the donor tissue facilitated allograft rejection but not in recipient mice. In addition, adoptive T cell transfer experiments in skin-grafted lymphopenic animals revealed a direct regulatory role for the α7nAChR on T cells. Taken together, our data demonstrate that the cholinergic pathway regulates alloreactivity and transplantation tolerance at multiple levels. One implication suggested by our work is that, in an organ transplant setting, deliberate α7nAChR stimulation of brain dead donors might be a valuable approach for preventing donor tissue inflammation prior to transplant.

## Introduction

Nonspecific immunosuppression through drug combinations remains the standard treatment to prevent allorejection for solid organ transplantation [1]. Strategies aiming to induce donor-specific transplantation tolerance have been experimentally developed [2], [3]. Donor-specific tolerance means that, in the absence of immunosuppression, potential graft-threatening alloreactivity is controlled while other immune responses remain intact (i.e. against cancers or infections) [4]. However, a body of evidence demonstrates that tolerance is often fragile and easily reversed, particularly in the context of tissue damage or infection [5], [6], [7], [8], [9], [10]. By interacting with “danger” receptors such as Toll-like receptors (TLRs), pathogen-associated molecular patterns (PAMPs) or endogenous damage-associated molecular patterns (DAMPs) trigger innate immune response, dendritic cell maturation and alloreactive T cell priming ultimately leading to allograft rejection. Indeed, several reports have shown that TLR-ligands [8], [11] or intact pathogens (such as *Listeria monocytogenesis* [8], [11]) can break allograft tolerance. Therefore, it is important to identify each process potentially prone to dampen immune storm at the innate or adaptive levels.

The cholinergic anti-inflammatory pathway has been identified as a natural regulatory system that prevents overwhelming of inflammation during sepsis to avoid irreversible organ damage [12]. During local inflammation, the sensory endings of the vagus nerve detect cytokines, DAMPS or PAMPS. In turn, efferent fibers of the vagus nerve trigger norepinephrine release by neurons in the celiac ganglion, allowing a specialized CD4+ T cell subtype in the spleen to synthesize acetylcholine (ACh) [12]. The ACh production by T cells dampens macrophage activation and pro-inflammatory cytokine release (also known as the anti-inflammatory reflex). In an endotoxemia model, vagus nerve stimulation (VNS) improves survival through the suppression of TNF-α and IL-1β release by macrophages [13]. The anti-inflammatory effect of ACh operates through the alpha7 nicotinic acetylcholine receptor (α7nAChR) expressed on macrophages. Indeed, α7nAChR-deficient (α7−/−) mice display heightened basal sensitivity to endotoxemia compared to wild-type animals and the protective effect of VNS is lost in α7−/− mice [13], [14]. The immunomodulatory effect of the cholinergic pathway has been initially described on innate components of inflammation such as macrophages [13], [15], [16], [17] and, more recently, several studies have focused on adaptive immune responses [13], [15], [16], [17]. Because T cells play a central role in the process of allograft rejection, each potential upstream or downstream regulatory mechanism should be carefully considered for inducing or maintaining transplantation tolerance. Importantly, deceased donors are brain dead, which, by definition, means an interruption of the cholinergic anti-inflammatory reflex favouring graft inflammation.

Herein, we investigated whether skin allograft rejection could be modulated by the cholinergic anti-inflammatory pathway. The capacity of female recipients to reject a male skin graft (minor mismatched combination) was compared in the presence or absence of the α7nAChR-dependent cholinergic pathway and after induction of local inflammation by TLR7 ligation. In this minor mismatched combination, female recipient CD4+ and CD8+ T cells recognize male peptides derived from the processing of the H-Y antigen and presented in class II or I major histocompatibility complex (MHC), respectively, by dendritic cells. H-Y antigen is a ubiquitous protein encoded by the Y chromosome. Since some discrepancies exist in the literature about the effect of an α7nAChR deficiency on T cells or dendritic cells [18], [19], [20], [21], we also compared the effect of a restricted α7nAChR deficiency on either the recipient T cell compartment or the non-T cell compartment including dendritic cells and keratinocytes.

## Materials and Methods

### Mice

Heterozygous α7nAChR (α7+/−) B6-129S7 mice were purchased from the Jackson Laboratory (Bar Harbor, Maine 04609 USA) and bred in our animal facility. The first generation (F1) α7+/+ or α7−/− animals were obtained from the same heterozygous parents and were distinguished through genotyping following JAX-protocol instructions. Therefore, the F1 α7−/− or control littermate mice (α7+/+) express rigorously the same genetic background. C57BL/6 Rag−/− mice were purchased from Jackson Laboratory (Bar Harbor, Maine 04609 USA) and bred in SPF (Specific Pathogen Free) facilities. 8 to 16 week-old animals were used for skin graft experiments. All animals received care in compliance with the Principles of Laboratory Animal Care formulated by the National Institute of Health (Guide for the Care and Use of Laboratory Animals, Eighth Edition, National Research Council, 2010) and all protocols were approved by the local committee for animal welfare - Comité d’Ethique du Biopole de Charleroi, Université Libre de Bruxelles.

### Skin Graft and T Cell Adoptive Transfer Experiments

Skin grafts were performed according to an adaptation of the method of Billingham and Medawar [22]. Mice were anesthetized with a mixture of 5% xylazine (ROMPUN®), 10% ketamine (KETAMINE®) in sterile saline 0.9% by intraperitoneal injection of 100 µl of the mixture/20 g body weight. Full-thickness donor tail skin (∼1 cm^2^) was grafted on the lateral flank of the recipient. Grafts were examined regularly after bandage removal on day 10 until day 30. Rejection was diagnosed when there was more than 75% epithelial breakdown. Where indicated, 250 mg TLR7 agonist, Imiquimod (ALDARA®, 3 M Pharmaceuticals) was applied at day 10 after transplantation and a second bandage was applied for an additional 5 days. In certain experiments, either α7+/+ or α7−/− males or females were used as skin graft donors. In adoptive transfer experiments, C57BL/6 Rag−/− female mice were reconstituted with intravenously injected 8×10^6^ CD90+ T cells purified from either α7+/+ or α7−/− female mice. CD90+ T cells were isolated from the spleen of either α7+/+ or α7−/− mice by positive selection using CD90.2 microbeads (Miltenyi Biotec) and an AutoMACS sorter (Miltenyi Biotec, Germany) following manufacturer instructions. Purity of CD90+ T cells was more than 90% as measured by flow cytometry analysis. One day after T cell transfer, mice were grafted with tail skin from male Rag−/− C57BL/6 mice.

### Histology

Seventeen days after transplantation, skin graft tissue was fixed for 24 hours in 10% formalin and embedded in paraffin. Histology was assessed on PAS-D stained 4 µm-thick sections.

### Polymerase Chain Reaction for mRNA Cytokines Measurements

Total RNA was extracted from lymph nodes and spleen using the MagnaPure LC RNA Isolation Kit III for tissue (Roche Diagnostics, Basel, Switzerland). Reverse transcriptase and real-time PCR were performed using Light Cycler-RNA Master Hybridization Probes (one-step procedure) on a Light cycler apparatus (Roche Diagnostics). Sequences for the PCR primers are: β-actin, forward, CCGAAGCGGACTACTATGCTA, reverse, TTTCTCATAGATGGCGTTGTTG, IL-2, forward, CCT-CAC-AGT-GAC-CTC-AAG-TCC, reverse, GG-GCT-TGT-TGA-GAT-GAT-GCT, IL-13, forward, GAG-CTG-AGC-AAC-ATC-ACA-CAA, reverse, CCC-TGT-GCA-ACG-GCA-GCA-TG, probe, GCC-AGG-TCC-ACA-CTC-CAT-AC, IFN-γ, reverse, GGA-TGC-ATT-CAT-GAG-TAT-TGG, forward, GCT-TCC-TGA-GGC-TGG-ATT-C, probe, TTT-GAG-GTC-AAC-AAC-CCA-CAG-GTC-CA, IL-17A, reverse, GCT-CCA-GAA-GGC-CCT-CAG, forward, CTT-TCC-CTC-CGC-ATT-GAC-A, probe, ACC-TCA-ACC-GTT-CCA-CGT-CAC-CCT-G (EUROGENTEC S.A.). β-actin was used as RNA loading control. For each cytokine RNA levels, cycle threshold (CT) was measured. Gene expression is expressed as Fold Increase (FI) by calculating FI = 2^−ΔΔCT^: ΔCT was determined by subtracting CT (β-actin) from CT (gene of interest) and ΔΔCT was calculated by subtracting ΔCT (control ungrafted mouse) from ΔCT (grafted mouse).

### Mixed Lymphocytes Reaction

Draining lymph node cells from skin-grafted and Imiquimod-treated recipients were harvested 30 days after transplantation for mixed lymphocyte culture. Irradiated spleen cells (2000 RAD) from female or male matched for α7nAChR expression or deficiency were used as stimulators (both stimulators and responders at the concentration of 5×10^6^ cells/ml). Cells were cultured in 48-well flat bottom plates (NUNC, Denmark), incubated at 37°C in a 5% CO_2_ atmosphere in culture medium (RPMI 1640 (BioWhittaker) each 500 ml supplemented with 2 mM L-glutamine, 1 mM non essential amino-acids, 5% FCS and 400 µl β-mercapto-ethanol). Supernatants were harvested after 24, 48 and 72 h of culture. Cytokine levels were measured by ELISA kits (Duoset; R&D Systems, Minneapolis). The detection threshold was 15 pg/ml for IL-2, IFN-γ and IL-17.

### Flow Cytometry

For T cell intracellular IL-17 and IFN-γ detection, graft draining lymph node cells were incubated in 48-well flat bottom plates (NUNC, Denmark) for 4 hours at 37°C in culture medium with PMA (50 ng/ml) and ionomycin (500 ng/ml) with brefeldin A (10 µg/ml) added for the last 2 hours. Then, cells were incubated with Fc blocking antibody (10′ at 4°C) and surface markers CD4 and CD8 were stained (20′ at 4°C). Cells were permeabilized with Perm/Wash buffer (BD Biosciences) and labeled with anti-cytokines antibodies (20′ at 4°C). Pacific Blue (PB)-conjugated rat anti-mouse CD8a (clone 53–6.7), APC-conjugated rat anti-mouse CD8a (clone 53-6.7), FITC-conjugated anti-mouse CD4 (clone L3T4GK1.5), PE-conjugated rat anti-mouse CD4 (clone RM4–5), FITC-conjugated rat anti-mouse IFN-γ (clone XMG1.2) were purchased from BD Pharmingen. APC-conjugated anti-mouse IL-17A (clone eBio17B7) was purchased from eBiosciences. PE-conjugated rat anti-mouse IL-2 (clone JES6-5H4) was purchased from SouthernBiotech. Flow cytometry analyses were performed on a CyAn-LX cytometer using Summit 4.1 software (DaKoCytomation).

### Bone Marrow-derived Dendritic Cell Culture

As previously described [23], bone marrow was flushed from femurs and tibiae of mice, filtered through nylon mesh and depleted of red blood cells after a short incubation in RBC lysis solution (ACK). RPMI 1640 culture medium was supplemented with 20 ng/ml recombinant mouse GM-CSF. At day 0, bone marrow progenitors were seeded in 6 well plates (10^6^ cells/well/4 ml culture medium). On day 3, 1 ml of fresh medium was added in each well. On day 6 and day 8, half of each supernatant is harvested and centrifuged, then cells were resuspended in 2 ml fresh medium. On day 10, all supernatants were collected and dendritic cells were incubated with 10 µg/ml Imiquimod (INVIVOGEN) for 36 hours. In some experiments, either nicotine (10, 100, 200, 500 µg/ml, SIGMA) or mecamylamine (2 µM, SIGMA) was added. Cytokine production was measured in supernatants collected after 36 hours using ELISA kit (R&D). Detection threshold was 32 pg/ml for TNF-α.

### Statistical Analysis

Graft survival curves were compared by the log-rank test. Other results were compared by using nonparametric Mann-Whitney two-tailed tests. P values <0.05 were considered as statistically significant.

## Results and Discussion

### The Cholinergic Pathway Modulates TLR-induced Skin Graft Rejection

**Figure 1:**
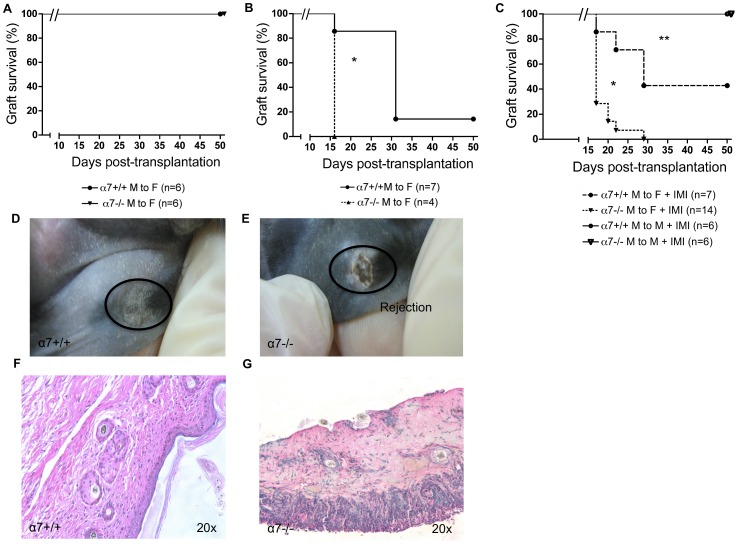
The cholinergic pathway modulates minor mismatched skin allograft rejection in the context of TLR stimulation. (**A** to **C**): Male to female (minor-mismatched) skin allografts were performed in either α7nAchR-sufficient (α7+/+) or α7nAchR-deficient (α7−/−) donor-recipient combinations. α7+/+ or α7−/− recipients are coming from the same heterozygous breeders (α7+/−) and genotyped (as described in Material and methods). Skin graft survival was compared in a specific pathogen free animal facility (SPF) (**A**) or in a conventional animal facility (**B**). In other experiments, male to male (matched) or male to female skin grafts were performed in the SPF animal facility. At day 10, 250 mg of imiquimod (a TLR7-agonist) was applied topically on the graft and 5 days later bandages were removed. Skin graft survival in either α7+/+ or α7−/− donor-recipient combinations is shown (**C**). Two independent experiments showed similar results, (*): p<0.05 compared to α7+/+ male to female; (**): p<0.05 compared to matched. (**D,E**): Macroscopic images of male skin grafts in female recipients 17 days after transplantation. A representative wild type (α7+/+) accepted (healthy) skin graft is shown in **D**. A rejected male skin graft in the α7−/− donor-recipient combination is shown in **E**. (**F,G**): Histology of imiquimod-induced minor mismatch skin graft rejection. The histology (20× magnification) of either accepted male skin in the α7+/+ donor-recipient combination (**F**) or α7−/− donor-recipient combination (**G**) is shown. Skin grafts were harvested 17 days after transplantation followed by PAS-D staining.

In order to investigate the effect of the cholinergic pathway on allograft rejection, minor-mismatched skin allografts were performed in either α7nAchR-sufficient (α7+/+) or α7nAchR-deficient (α7−/−) donor-recipient combinations. Graft survival was compared between male α7+/+ donors to female α7+/+ recipients and male α7−/− donors to female α7−/− recipients. In a specific pathogen-free (SPF) animal facility, allografts were tolerated similarly in both groups (Fig. 1A). However, this state of spontaneous graft acceptance was broken by keeping mice in a conventional non-barrier animal facility, with a significantly faster rejection in the α7−/− group compared to wild type (Fig. 1B). This difference suggests that the cholinergic pathway regulates one or more mechanisms of innate and adaptive immune responses in the context of immune stimulation by unknown pathogens present in the non-barrier facility. Of note, parvovirus was detected in sentinel mice housed in other rooms in the conventional animal facility at the time of our experiments. Parvovirus infection in mice has been previously described to accelerate skin allograft rejection [24]. Many recent studies focused on the impact of either infection or TLR-agonists (PAMPS) on allograft tolerance [6], [8], [25]. One mechanism by which viruses or other pathogens can reverse allograft acceptance relies on TLR activation.

In order to mimic the effect of PAMPS on skin allograft rejection in a more controlled setting, we tested the effect of the TLR7/8 agonist, imiquimod, in the SPF animal facility. TLR7 stimulation by imiquimod has been reported to locally enhance skin inflammation and alloreactive T cell recruitment [26]. As shown in Fig. 1C, topical imiquimod application for 5 days after bandage removal restored α7−/− male skin graft rejection by α7−/− female recipients. Up to 40% of α7+/+ male skin grafts remained intact on α7+/+ recipients until day 30 whereas all skin grafts were rapidly rejected in the α7−/− group. Of note, imiquimod did not induce rejection of isogenic grafts (male to male) in either α7+/+ or α7−/− groups. Histological analysis (Fig. 1D to 1G) revealed destroyed architecture in the rejected α7−/− skin grafts together with a dense inflammatory cell infiltrate and necrosis while remaining α7+/+ skin grafts showed normal histology. Again, this indicates that the cholinergic pathway is able to modulate the loss of tolerance induced by TLR7 activation.

### The Absence of the Cholinergic Pathway Results in Enhanced T Cell Alloreactivity

**Figure 2:**
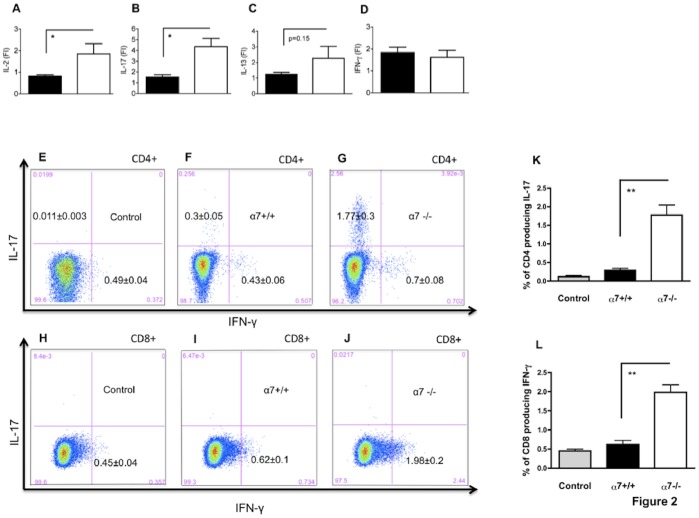
The cholinergic pathway dampens immune responses in the draining lymph node of skin-grafted and imiquimod-treated female recipients. (**A** to **D**): From **A** to **D**, mRNA coding for IL-2, IL-17, IL-13 and IFN-γ were quantified by qPCR in draining lymph nodes 30 days after transplantation. Black bars represent wild type donor-recipient combination and white bars the α7−/− donor-recipient combination. Results are expressed as fold increase (FI = 2^−ΔΔCT^) of quantities measured in lymph nodes from either ungrafted α7+/+ or α7−/− mice, n = 4 to 8 mice per group. (*): p<0.05 compared with wild type α7+/+ donor-recipient combination. (**E** to **G**): Intracellular IL-17 production in CD4 gated T cells after 4 hours of PMA-ionomycin stimulation is shown in control α7+/+ ungrafted mice (**E**), wild type donor-recipient combination (**F**) and α7−/− donor-recipient combinations (**G**). (**H** to **J**): Intracellular IFN-γ production in CD8 gated T cells after 4 hours of PMA-ionomycin stimulation is shown in control α7+/+ ungrafted mice (**H**), in wild type donor-recipient combination (**I**) and α7−/− donor-recipient combinations (**J**). (**K,L**): Bars summarize the percentages of IL-17+ CD4 T cells (**K**) and IFN-γ+ CD8 T cells (**L**) in either the wild type (black bars) or the α7−/− (white bars) donor-recipient combination. Controls have been harvested from α7+/+ ungrafted mice (grey bars). n = 4 to 8 mice/group. No difference regarding IL-17 (Supplementary figure 1) or IFN-γ production has been observed between α7+/+ and α7−/− ungrafted controls. (**): p<0.01 α7−/− compared to α7+/+ donor-recipient combinations.

We next examined T cell responses occurring in graft draining lymph nodes during imiquimod-induced rejection in α7+/+ and α7−/− mice. Draining lymph nodes were harvested 30 days after skin transplantation and mRNA coding for IL-2, IL-13, IL-17 and IFN-γ were quantified. As shown in Fig. 2A and B, levels of IL-2 mRNA and IL-17 mRNA were significantly higher in draining lymph nodes in the α7−/− group compared to the α7+/+ group. IL-13 mRNA production tended to be higher in the α7−/− group though the difference did not reach statistical significance (Fig. 2C), and no difference was detected for IFN-γ mRNA levels (Fig. 2D). In parallel, we performed intracellular cytokine staining in lymph node T cells after a short PMA-ionomycin stimulation. We observed an increased number of IL-17 producing CD4+ T cells among lymph nodes from α7−/− mice compared to α7+/+ mice (Fig. 2E to G) and it has been quantified in Fig. 2K. Although no difference of IFN-γ mRNA production was detected by PCR, a higher percentage of IFN-γ positive CD8+ T cells (but not CD4+ T cells) was observed in the α7−/− group compared to the α7+/+ group (Fig. 2H to J) and it has been quantified in Fig. 2L. No difference regarding IL-17 (Fig. S1) or IFN-γ intracellular production was noticed when α7−/− ungrafted mice were compared with α7+/+. Of note, the persisting male skins in the α7+/+ group might influence T cell alloreactivity compared with the α7−/− group in which all skins were rejected at day 30. Nevertheless, T cell response was enhanced in recipients that lost their graft (the α7−/− group), rather suggesting a T cell priming by male antigens.

**Figure 3:**
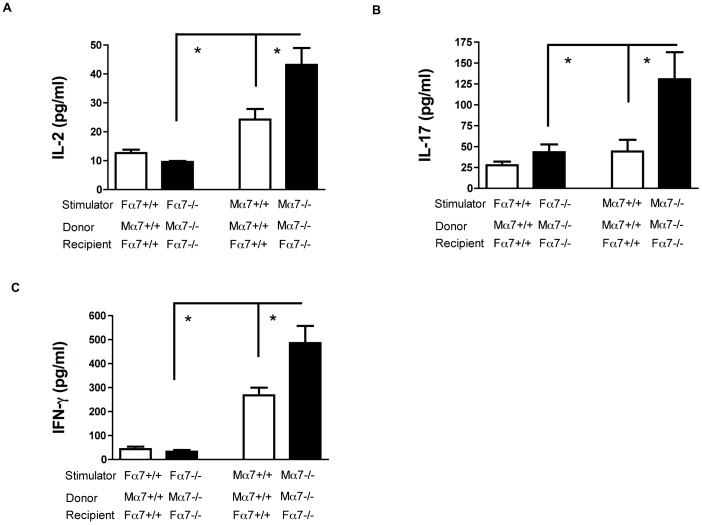
Anti-donor T cell responses are regulated through the cholinergic anti-inflammatory pathway. (**A** to **C**): Splenocytes harvested from either α7+/+ (white bars) or α7−/− (black bars) male to female donor-recipient combinations were stimulated in mixed lymphocyte reactions with α7nAchR-matched female or male splenocytes. IL-2 (**A**), IL-17 (**B**) and IFN-γ (**C**) production in the supernatants was quantified by ELISA. (F) indicates female and (M) indicates male. (*): p<0.05 compared with wild type α7+/+ donor-recipient combination or female stimulation.

In order to compare T cell alloreactivity between α7+/+ and α7−/− groups in an alloantigen-specific manner, recipient splenocytes were stimulated in a mixed lymphocyte reaction (MLR). Splenocytes from the α7−/− group or the α7+/+ group were stimulated with either α7-nAChR-matched irradiated female (autologous) or male (allogeneic) spleen cells (Fig. 3+Fig. S2). MLR supernatants were harvested 48 or 72 hours after stimulation and cytokines were quantified by ELISA. Autologous stimulation did not trigger significant IL-2, IL-17, or IFN-γ production. For allogeneic stimulation, α7−/− splenocytes (black bars) released significantly higher levels of IL-2 (Fig. 3A), IL-17 (Fig. 3B) and IFN-γ (Fig. 3C) compared to α7+/+ splenocytes (white boxes). Control groups in mixed lymphocyte reaction are stimulated splenocytes from ungrafted mice (Fig. S2). Altogether, these results demonstrate that the cholinergic anti-inflammatory pathway blunts the Th17 and CD8 T cell alloreactivity after TLR7 activation.

**Figure 4:**
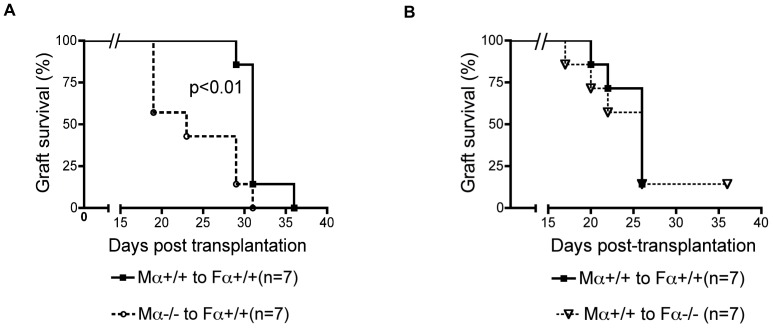
Comparison of selective α7nAChR deficiencies in donors or recipients. (**A**): α7+/+ wild type female mice were grafted with either α7+/+ or α7−/− male skin grafts. Imiquimod was applied from day 10 to day 15 after transplantation. Graft survival is compared. (**B**): Graft survival of male α7+/+ skin grafts on either α7+/+ or α7−/− female recipients treated with imiquimod from days 10 to 15.

Whether the cholinergic pathway acts directly on effector T cells or indirectly through the modulation of afferent inflammatory stimuli (such as dendritic cell activation by TLR7) was the next question that we addressed. First, we tested the importance of α7nAChR expression in donor tissue cells by grafting female recipients with mismatched skin harvested from either α7nAChR-deficient or wild type male mice followed by imiquimod treatment. As shown in Fig. 4A, α7−/− skin grafts were rejected significantly faster than α7+/+ skin grafts in α7+/+ recipients, although the kinetics were slightly slower than when both donor and recipient are lacking α7nAChR (α7−/− group, Fig. 1C). In contrast, α7+/+ skin grafts were rejected in α7−/− recipients and α7+/+ recipients with no significant difference in the kinetics (Fig. 4B). This demonstrates that the major role of α7nAChR in regulating allorejection requires expression on donor cells, such as keratinocytes and dendritic cells which are in contact with the TLR7 agonist.

To address whether α7nAChR activation on donor-derived dendritic cells could affect cytokine secretion, we measured the effects of nicotine, an α7nAChR agonist on TNF-α production stimulated by TLR7 activation. We compared TNF-α production by imiquimod-stimulated dendritic cells derived from either wild type or α7nAChR-deficient mice in the presence of increased doses of nicotine (Fig. S3). The anti-inflammatory effect of nicotine was partially reversed by the α7nAChR antagonist (mecamylamine) and was not significant with dendritic cells derived from α7nAChR-deficient mice. This experiment confirmed the capacity of the cholinergic pathway to dampen, in a dose dependent manner, the production of TNF-α, a proinflammatory cytokine.

**Figure 5:**
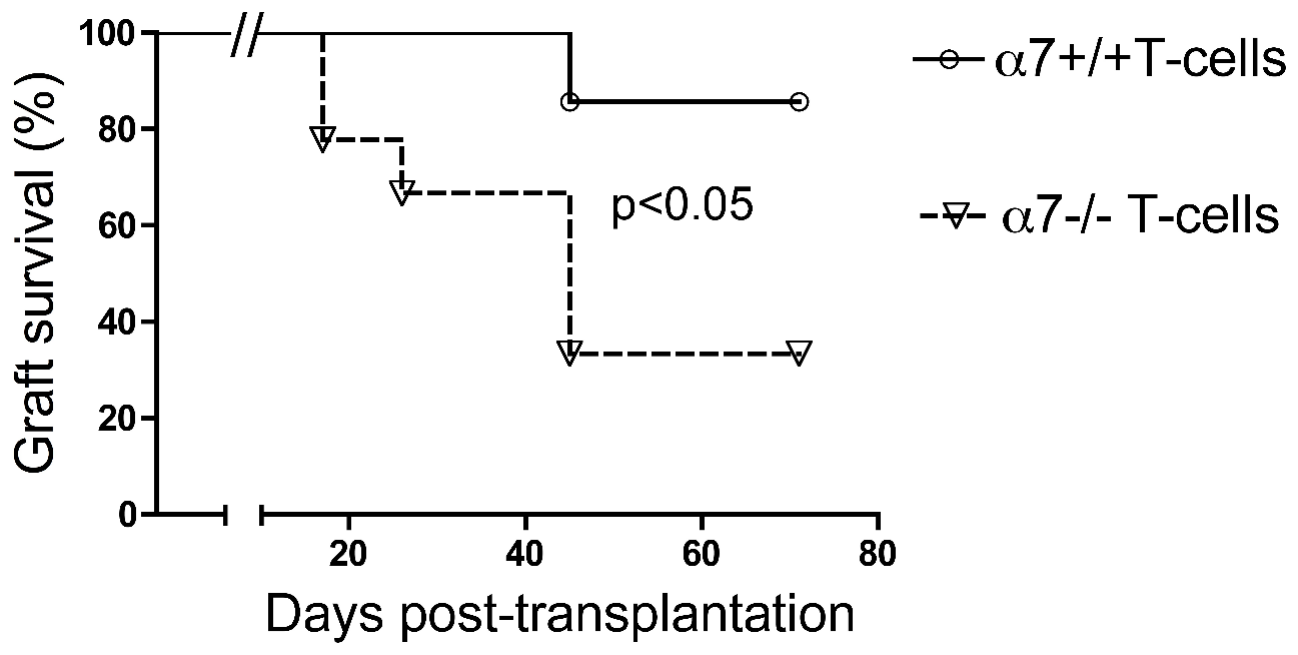
Importance of the α7nAChR on T cells in imiquimod-induced skin allograft rejection. Lymphopenic female Rag−/− mice were reconstituted with 8×10^6^ CD90+ T cells (containing both CD4+ and CD8+ T cells) purified from either α7+/+ or α7−/− female mice. Reconstituted mice were transplanted with a male skin graft harvested from male Rag−/− mice and imiquimod was applied for days 10 to 15. Skin graft survival in both conditions is shown.

We next investigated whether α7nAChR expression on T cells directly modulated alloreactivity. For this purpose, Rag−/− female recipients were adoptively transferred with CD90+ T cells (including both CD4+ and CD8+ T cells) purified from either α7nAChR-deficient or wild type mice. One day after T cell transfer, mice were grafted with tail skins from male Rag−/− donors, followed by repeated imiquimod applications. As expected, the levels of rejection were lower and the rate of rejection was slower in reconstituted mice (Fig. 5) compared with mice with normal T cell levels (Fig. 1C). Interestingly, α7nAChR-deficient T cell transfer induced significantly more rejections compared to wild type T cells (Fig. 5). This result demonstrates that α7nAChR can regulate T cell alloreactivity directly.

Our results show that the spontaneous acceptance of minor mismatched skin allografts is abrogated by either a local TLR7 agonist application or a pathogen-contaminated environment. The observation that TLR activation reverts allograft acceptance is consistent with previous studies [6], [8], [11], [25] and provided us the opportunity to test the impact of the cholinergic anti-inflammatory pathway in this setting. TLR activation on dendritic cells enhances alloantigen presentation to T cells promoting Th1, Th2, or Th17 responses depending on the cytokine environment and the type of TLR involved [5], [8], [11], [27]. We took advantage of established models using the TLR7 agonist imiquimod to trigger T cell-dependent skin lesions as described in a GVHD model [26] and psoriasis [27]. We followed a protocol similar to the psoriasis study [27] which showed that daily application of imiquimod promotes skin infiltration by dendritic cells, macrophages and neutrophils through an IL23/IL17 axis. In our study, we showed that the full donor/recipient deficiency or even a single donor deficiency for the α7nAChR promoted TLR7-induced mechanisms of allograft rejection. Like TLR7, α7nAChR is expressed by dendritic cells, including Langerhans cells in the skin, keratinocytes and endothelial cells [17], [28], [29]. Activation of α7nAChR on macrophages by nicotine or other nicotinic agonists reduces the production of TNF-α, IL-1β and HMGB-1 by a suppression of NF-κB activation [14], [30]. On activated endothelial cells, this cholinergic pathway reduces expression of adhesion molecules necessary for inflammatory infiltration [15]. These effects could explain a local protective effect of the cholinergic pathway by preventing effector recruitment even when immune effector cells express functional α7nAChR (like in the α7nAChR-deficient donor to wild type recipient combination). On the other hand, by dampening dendritic cell activation and full maturation, α7nAChR activation could also prevent optimal T cell priming which is in agreement with the higher T cell response we observed in MLR with T cells from α7nAChR-deficient mice compared to wild type mice. Indeed, α7nAChR activation by nicotine promotes the development of semi-mature DC with less co-stimulatory molecule expression (CD80) and MHC II, reduced capacity for antigen uptake and IL-12 release [18], [19], which is in line with our own observation of reduced TNF-α production by imiquimod-stimulated dendritic cells in the presence of nicotine. Expression of α7nAChR on donor-derived cells is partly responsible for regulating T cell alloreactivity, as shown in our experiments using α7nAChR-deficient donors on wild type recipients. However, because both donor and recipient dendritic cells process and present male antigen-derived peptides to female T cells [31], [32], [33], α7nAChR expression on donor dendritic cells cannot completely explain the results we observed in the knock-out to wild type combination. Therefore, the impact of α7nAChR deficiency should be interpreted beyond dendritic cells.

From the T cell point of view, our results can be summarized as follows: the global alloreactive T cell response (CD4+ and CD8+) was enhanced in the absence of both donor and recipient α7nAChR and a T cell-restricted α7nAChR-deficiency enhanced allograft rejection. Usually, the inflammatory reflex involves norepinephrine release by the splenic nerve followed by acetylcholine synthesis and release by a small CD44^high^ CD62L^low^ CD4+ T cell subset controlling macrophages and potentially other cells expressing the α7nAChR [12], [16]. This paradigm is compatible with the enhanced T cell response we observed in the full α7nAChR-deficient combination by preventing the inflammatory reflex circuit downstream of the acetylcholine-producing T cells. Nevertheless, this is not supported by the unaffected kinetics of rejection we observed in α7nAChR-deficient mice receiving skin grafts from wild type mice most probably because of a relatively mild effect of the α7nAChR on recipient T cells. This effect became apparent only in adoptive T cell transfer experiments in Rag−/− mice. In these experiments, the rejection kinetics was delayed compared with T cell-repleted animals (due to the very small number of precursors), which allowed to reveal a regulatory role of the α7nAChR expression on T cells. The cholinergic effect on T cells is complex. Brenner et al. observed that nicotine administration decreased T cell proliferation and Th1 and Th17 cytokine production in response to encephalitogenic antigen and reduced the clinical severity of experimental autoimmune encephalomyelitis (EAE) [20]. Activation of α7nAChR on wild type T cells reduced NF-κB transcription, while α7nAChR-deficient T cells displayed a complex phenotype with spontaneous reduced proliferation and cytokine production. Accordingly, EAE was less severe in α7nAChR-deficient mice. In an arthritis model, α7nAChR-deficient mice display milder arthritis and less cartilage destruction and again T cells were less responsive to conA [21]. Our results of adoptive transfer experiments show, for the first time, an intrinsic *in vivo* effect of α7nAChR on T cells. This inhibitory effect is consistent with the previous studies of α7nAChR in T cell-mediated disease models.

A recent study has described modifications of the cholinergic pathway after brain death and resuscitation [34]. Cerebral ischemia results in a significant decline in central cholinergic activity and in the inflammatory reflex. Accordingly, we might suspect that brain dead donors are unable to maintain central cholinergic activity and the inflammatory reflex before organ harvesting and ischemia reperfusion. Further investigations will be necessary to attest the importance of the cholinergic anti-inflammatory pathway in this clinical setting.

## Supporting Information

Figure S1 **Intracellular IL-17 production by CD4 T cells in control mice.** (**A**,**B**) Percentage of CD4 T cells producing intracellular IL-17 in control α7+/+ ungrafted mice (**A**) and control α7−/− ungrafted mice are shown (**B**). (**C**) Bars summarize the amount of IL-17+ CD4 T cells in α7+/+ ungrafted mice (black bar) and in control α7−/− ungrafted mice (white bar). There is no statistical difference between groups (n = 4 mice/group).
(TIFF)

Figure S2 **Control groups in mixed lymphocyte reaction.** (**A** to **C**): Splenocytes harvested from either α7+/+ (white bars) or α7−/− (black bars) ungrafted mice (called controls) were stimulated in mixed lymphocyte reactions with α7nAchR-matched female or male splenocytes. IL-2 (**A**), IL-17 (**B**) and IFN-γ (**C**) productions in the supernatants were quantified by ELISA. (F) indicates female and (M) indicates male.
(TIFF)

Figure S3 **Nicotine suppresses TNF-α production in bone marrow dendritic cells derived from α7+/+ but not α7−/− mice after imiquimod stimulation.** TNF-α productions in bone marrow-derived dendritic cells (BMDC) from either α7+/+ or α7−/− mice are shown. BMDC were isolated from female mice and were stimulated with imiquimod (10 µg/ml). Nicotine (0,10,100 µg/ml) and/or mecamylamine (2 µM) were added simultaneously. TNF-α productions in the supernatant were measured by ELISA 36 hours after stimulation. Bars on the left represent α7+/+ BMDC whereas bars on the right represent α7−/− BMDCs. TNF-α production from unstimulated BMDC (no imi no nic) without imiquimod and without nicotine is under the detection level of 32 pg/ml (det.level). (*): p<0.05 between conditions with and without nicotine in α7+/+ animals. Results are representative of three independent experiments.
(TIFF)
