# Supplementary figures and images for: The Cholinergic Anti-Inflammatory Pathway Delays TLR-Induced Skin Allograft Rejection in Mice: Cholinergic Pathway Modulates Alloreactivity
Source: PLoS One. 2013 Nov 21;8(11):e79984. doi: 10.1371/journal.pone.0079984 (PMC3836989; doi:10.1371/journal.pone.0079984)

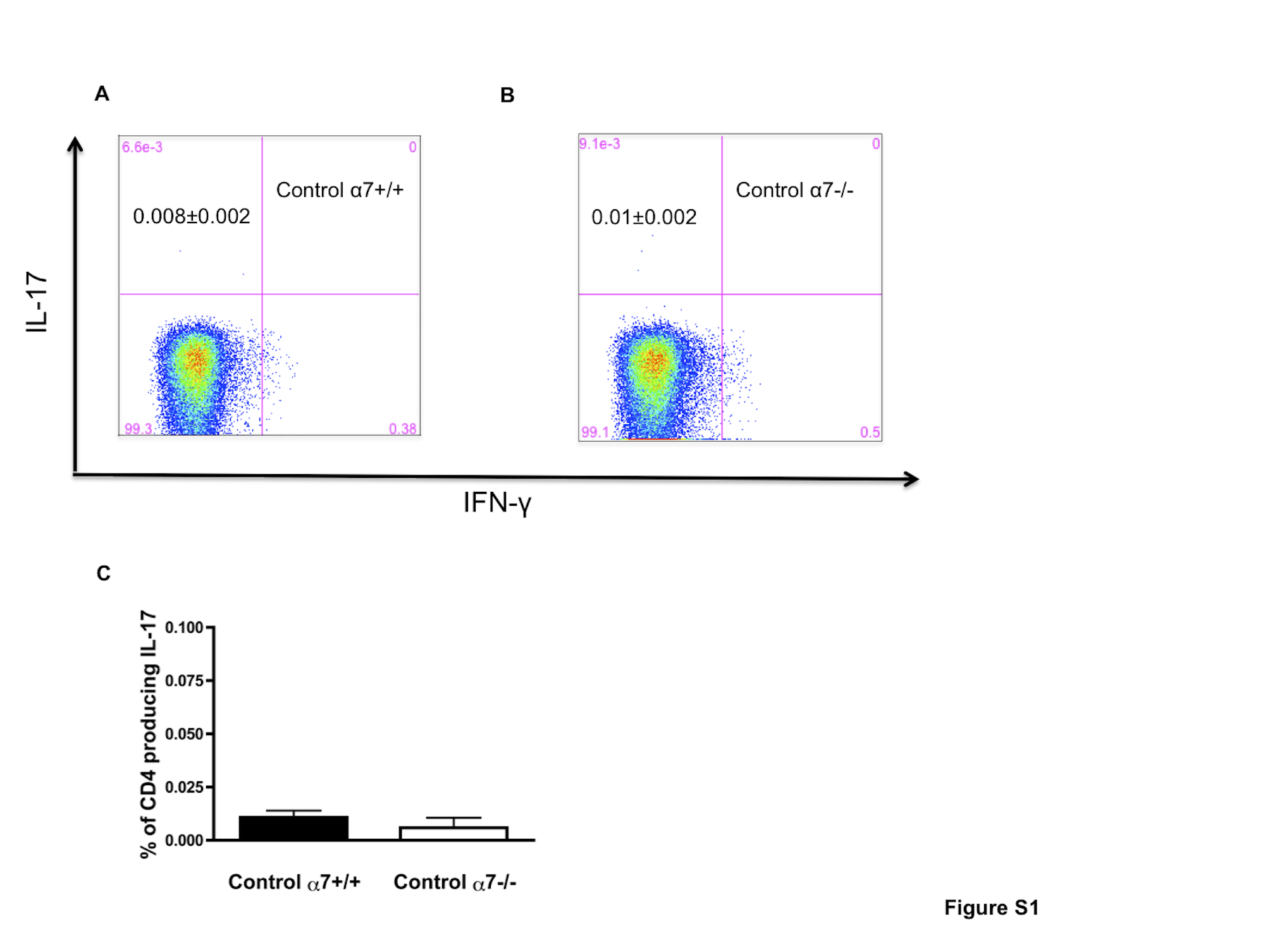

Supplement: Figure S1 — Intracellular IL-17 production by CD4 T cells in control mice. (A,B) Percentage of CD4 T cells producing intracellular IL-17 in control α7+/+ ungrafted mice (A) and control α7−/− ungrafted mice are shown (B). (C) Bars summarize the amount of IL-17+ CD4 T cells in α7+/+ ungrafted mice (black bar) and in control α7−/− ungrafted mice (white bar). There is no statistical difference between groups (n = 4 mice/group). (TIFF) [file pone.0079984.s001.tiff]

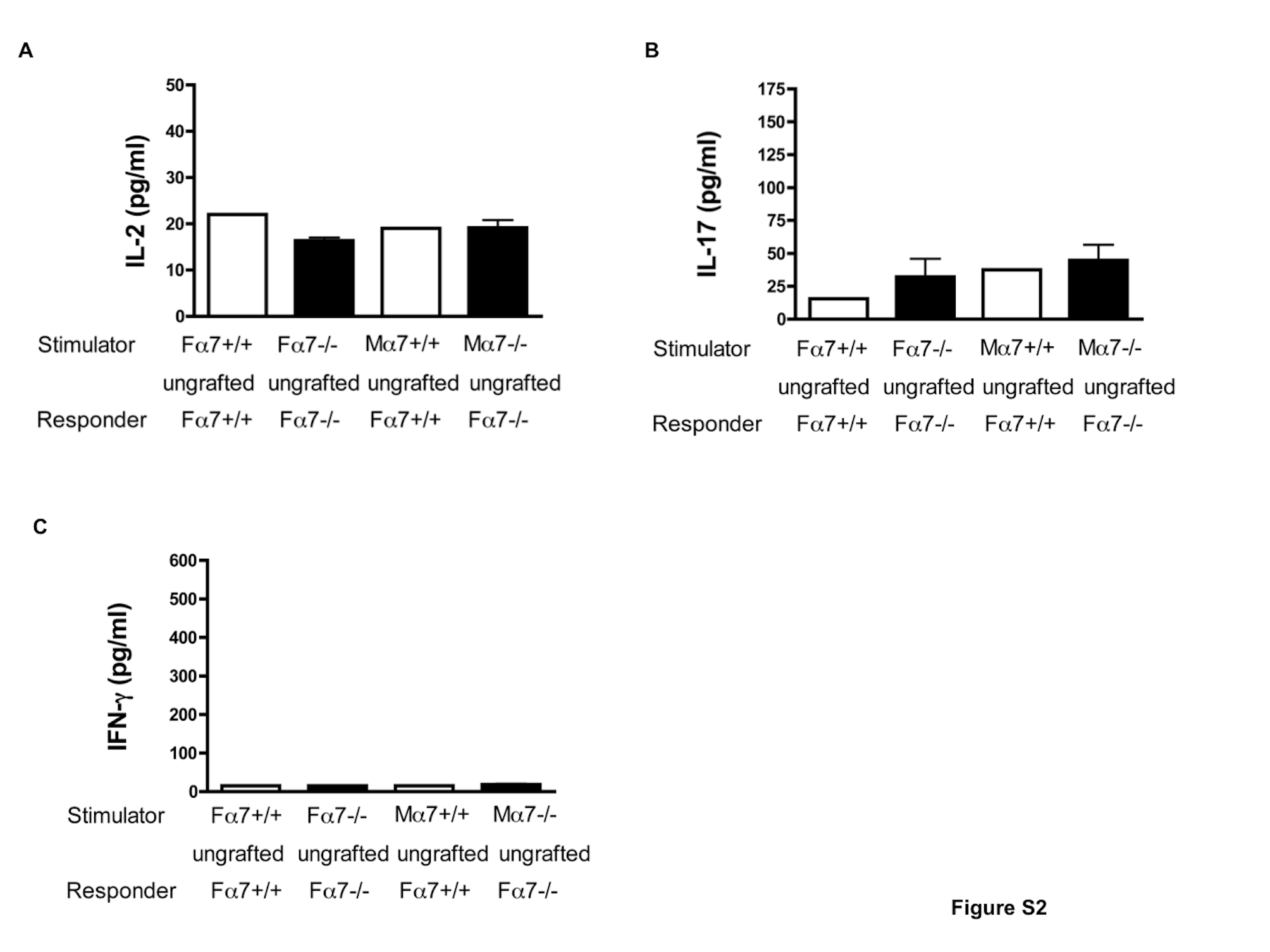

Supplement: Figure S2 — Control groups in mixed lymphocyte reaction. (A to C): Splenocytes harvested from either α7+/+ (white bars) or α7−/− (black bars) ungrafted mice (called controls) were stimulated in mixed lymphocyte reactions with α7nAchR-matched female or male splenocytes. IL-2 (A), IL-17 (B) and IFN-γ (C) productions in the supernatants were quantified by ELISA. (F) indicates female and (M) indicates male. (TIFF) [file pone.0079984.s002.tiff]

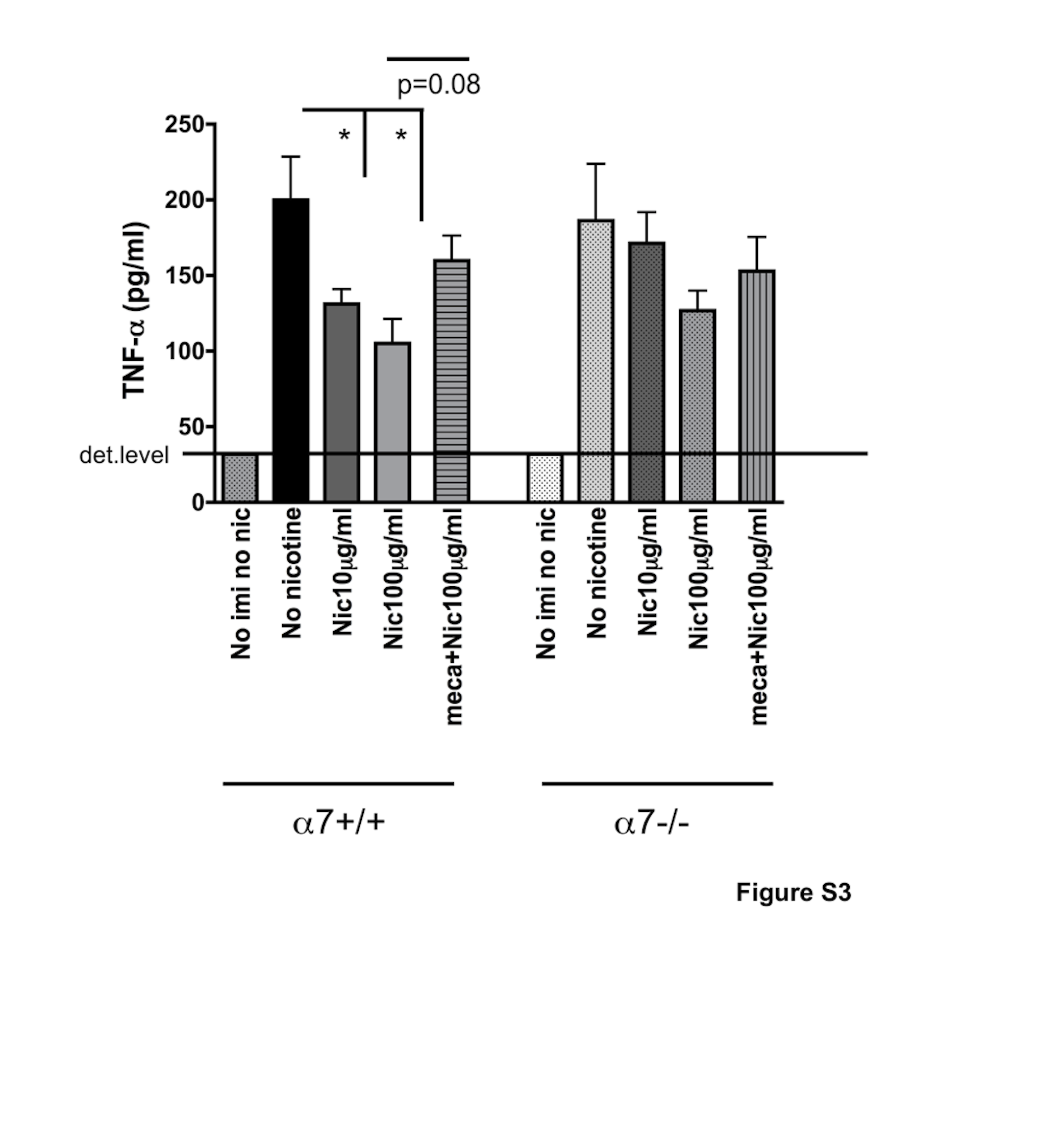

Supplement: Figure S3 — Nicotine suppresses TNF-α production in bone marrow dendritic cells derived from α7+/+ but not α7−/− mice after imiquimod stimulation. TNF-α productions in bone marrow-derived dendritic cells (BMDC) from either α7+/+ or α7−/− mice are shown. BMDC were isolated from female mice and were stimulated with imiquimod (10 µg/ml). Nicotine (0,10,100 µg/ml) and/or mecamylamine (2 µM) were added simultaneously. TNF-α productions in the supernatant were measured by ELISA 36 hours after stimulation. Bars on the left represent α7+/+ BMDC whereas bars on the right represent α7−/− BMDCs. TNF-α production from unstimulated BMDC (no imi no nic) without imiquimod and without nicotine is under the detection level of 32 pg/ml (det.level). (*): p<0.05 between conditions with and without nicotine in α7+/+ animals. Results are representative of three independent experiments. (TIFF) [file pone.0079984.s003.tiff]
